# Non-prostate uptake on ^18^F-PSMA-1007 PET/CT: a case of myeloma

**DOI:** 10.1259/bjrcr.20200102

**Published:** 2020-11-03

**Authors:** Sowmya Veerasuri, Stewart Redman, Richard Graham, Chris Meehan, David Little

**Affiliations:** 1 Department of Radiology, Royal United Hospital, Combe Park, Bath, Avon, United Kingdom; 2 Department of Cellular Pathology, Royal United Hospital, Combe Park, Bath, Avon, United Kingdom

## Abstract

Prostate-specific membrane antigen (PSMA), a glycoprotein that is highly expressed in prostate cancer, has been used as a target for molecular radiotherapy as well as imaging. Over the last couple of years, ^18^F-PSMA gained popularity due to its longer half-life (110 min) compared to gallium ^68^Ga-PSMA (68 min). This has helped the dissemination beyond large metropolitan centres. In addition, due to the low background activity in the urinary bladder (1.2% injected dose over 2 h compared to 10% injected dose over 2 h for ^68^Ga), ^18^F-PSMA helps detect local recurrence or spread to pelvic nodes more readily as lesions are not masked by physiological urinary excretion.

Despite excellent sensitivities of PSMA positron emission tomography modalities, it is noteworthy that PSMA expression is not specific to the prostate. A variety of normal tissues express PSMA with intense uptake noted in salivary glands, lacrimal glands, the liver, spleen, pancreas, small intestine, bladder and renal cortex.

In this case report, we describe an example of non-prostatic PSMA uptake in a patient imaged with ^18^F-PSMA-1007 positron emission tomography/CT that showed an avid lytic lesion in manubrium. The patient was subsequently proven by biopsy to have myeloma. Our case report illustrates a potential pitfall when imaging patients with ^18^F PSMA-1007 and adds to the growing body of literature of non-prostatic uptake of PSMA and highlights the need for reporters to be aware of this uptake.

## Introduction

Prostate cancer is the commonest cause of death in males in the UK. Imaging plays an important part not only in establishing initial diagnosis but also to evaluate response to treatment and assess for recurrence. The last decade has seen significant improvements in imaging techniques, especially with the development of positron emission tomography (PET). Various tracers have been developed and breakthroughs obtained with ligands targeting the prostate-specific membrane antigen (PSMA). Since 2011, Gallium-based ligand (^68^Ga-PSMA)^1^ PET has been increasingly used and has shown excellent detection rates even at low prostate-specific antigen (PSA) levels, establishing its role in evaluating biochemical recurrence (BCR) following radical prostatectomy/radiotherapy. However, due to the uptake in bladder, local recurrence has been hard to detect.^2^ Therefore, there has been ongoing research for other PSMA based ligands and in 2016 Fluorine-based PSMA ligand, ^18^F-PSMA 1007 was developed.^3^ Since then, there has been increasing use of this ligand for assessing BCR after prostatic cancer therapy and results have been promising. Whilst PSMA-based ligands have been shown to sensitive, non-prostatic pathological uptakes have been reported. Most of the current understanding of the non-prostatic uptake is from ^68^Ga-PSMA PET^4^ and only a handful of reports are in cases of ^18^F-PSMA 1007. In this case report, we describe an example of non-prostatic PSMA uptake in a patient imaged with ^18^F-PSMA-1007 PET/CT that showed a lytic lesion in manubrium, which was subsequently proven to be myeloma on biopsy.

## Case history

A 69-year-old male patient with no significant past medical history was diagnosed with prostate cancer in 2016. His initial staging was T3b G7 (4 + 3) with seminal vesicle extension and extracapsular invasion. He underwent prostatectomy in 2016 and subsequent prostate bed radiotherapy a few months later. He also had a short course of bicalutamide in 2017.

## Investigations

His initial post-treatment PSA was undetectable, but was 0.6 ng ml^−1^, 1 year later. His blood tests revealed slowly increasing PSA in 2018 which led to further imaging. ^18^F Choline PET scan was performed in June 2018 and this showed no local recurrence or metastatic disease. His PSA continued to rise and was 4.4 ng ml^−1^ in 2019.

**Figure 1.**
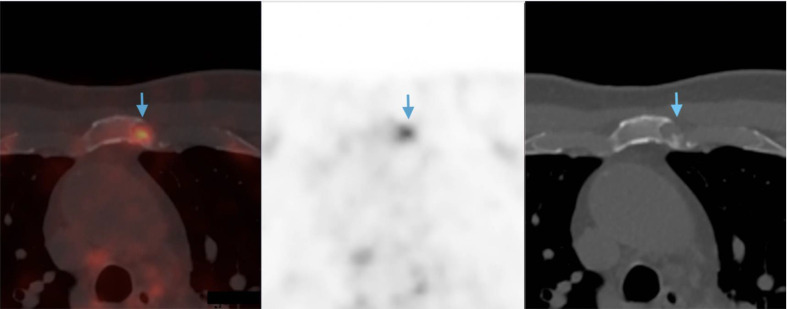
Selected images from the PSMA PET showing mild uptake in the manubrial lesion (blue arrows) (SUVmax of lesion: 6.1) and the CT equivalent showing lytic bone lesion. PET, positron emission tomography; PSMA, prostate-specific membrane antigen; SUVmax, maximum standardised uptake value.

**Figure 2.**
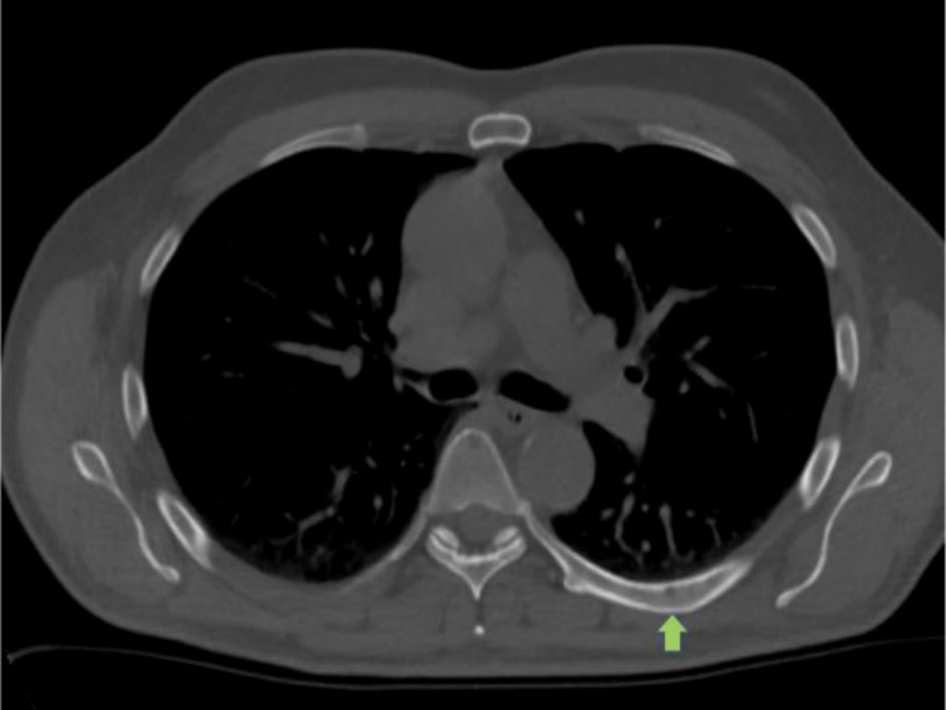
3 mm lucent lesion in the posterior left seventh rib highlighted by green arrow.

**Figure 3.**
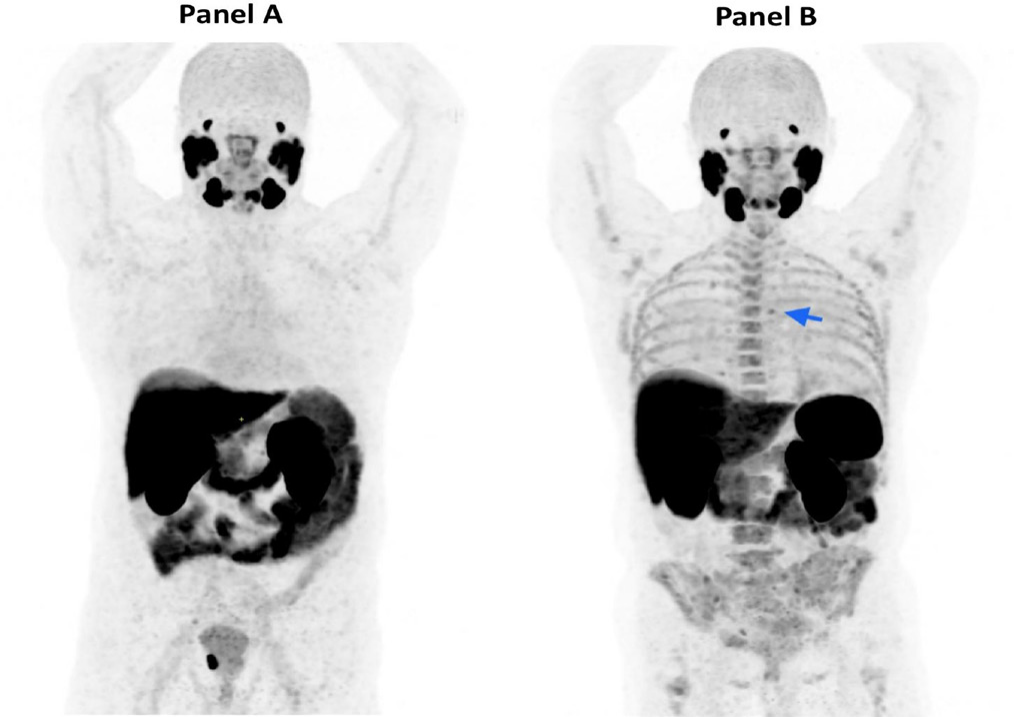
Panel A: MIP showing normal PSMA PET marrow uptake and also focal uptake in the right side-of prostate. Also note normal PSMA uptake pattern in salivary glands, lacrimal glands, the liver and renal cortex. Panel B: MIP showing our patient with diffuse marrow uptake (SUVmax scale 0–10 g ml^−1^; SUVmax of Lumbar spine: 5.3 g ml^−1^) and focal lesion (blue arrow). MIP, maximum intensity projection; PET, positron emission tomography; PSMA, prostate-specific membrane antigen; SUVmax, maximum standardised uptake value.

An ^18^F-PSMA PET/CT was performed in December 2019 to further investigate the source of the BCR. A mildly avid lytic lesion was seen in the manubrium (Figure 1). A further 3 mm lucent lesion was seen in the left seventh rib (Figure 2) along with mild diffuse marrow uptake (Figure 3). The pattern of diffuse marrow uptake and the lytic lesion were thought to be unusual in prostate cancer metastases and a suspicion of alternate pathology was raised, with myeloma the most likely differential.

**Figure 4.**
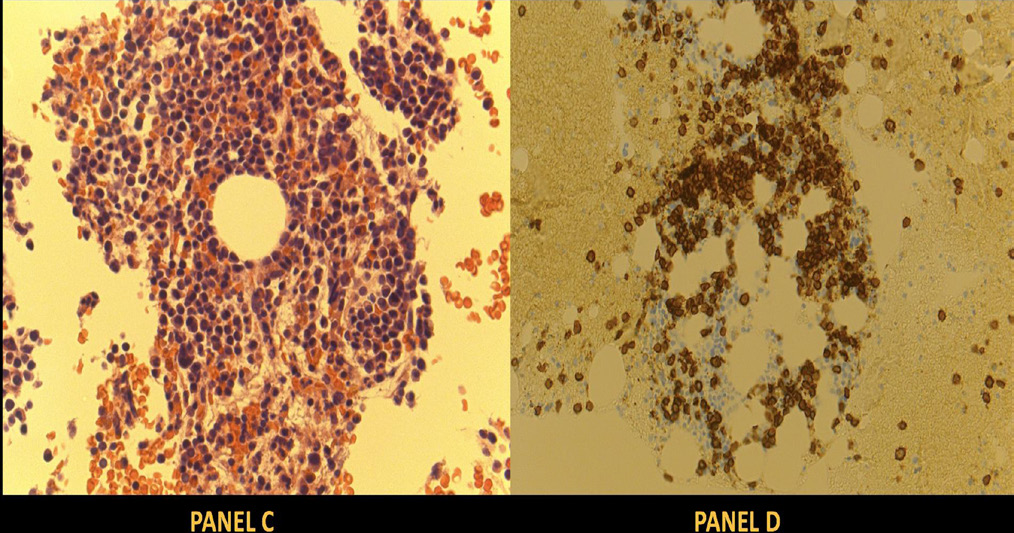
H&E stain showing numerous plasma cells in the trephine with few background cells. (x40) (Panel C). CD 138 immunohistochemistry confirming plasma cells. (x40) (Panel D).

The patient underwent a CT-guided fine needle aspiration (FNA) of the lytic manubrium lesion as core biopsy was not possible due to the small size of lesion. The FNA was non-diagnostic. A myeloma screen was performed with electrophoresis, which detected presence of significant excess of κ light chains (KLR 451). Bone biopsy confirmed κ light chain myeloma (Figure 4). He is currently undergoing treatment for myeloma under the care of Haematologists.

## Discussion

PET/CT using Fludeoxyglucose (^18^F) (FDG) is routinely used to stage and assess treatment response in many common cancers. However, due to low metabolism in prostatic cancer, FDG PET/CT has low yield. Other tracers have been developed in the imaging of prostate cancer including; ^68^Ga-PSMA, ^18^F-PSMA, ^18^F-fluoromethylcholine (^18^FMH), ^18^F-fluoroethylcholine (^18^FCH), anti1-amino3 ^18^F-fluorocyclobutane-1carboxylic acid (F-fluviclovine), and ^11^C-choline.

PSMA, a glycoprotein that is highly expressed in prostate cancer, has been used as a target for molecular radiotherapy as well as imaging. ^68^Ga-PSMA PET/CT has been shown to be effective in identifying the site of disease in patients with BCR due to its high sensitivity and specificity.^5^ Logistical challenges and the need for a ^68^Ga generator have limited the widespread use of ^68^Ga-PSMA and fluorinated PSMA tracers with a longer half-life have been developed. Over the last couple of years, ^18^F-PSMA gained popularity due to its longer half-life (110 min) compared to ^68^Ga-PSMA (68 min). This has helped the dissemination beyond large metropolitan centres. In addition, due to the low background activity in the urinary bladder (1.2% injected dose over 2 h compared to 10% injected dose over 2 h for ^68^Ga),^6^ ^18^F-PSMA helps detect local recurrence or spread to pelvic nodes more readily as lesions are not masked by physiological urinary excretion.^2^

CT scan, although initially used extensively, as it is readily available, is not considered a good modality in monitoring recurrence of prostatic carcinoma. CT may be a good aid for assessing nodal and distant metastases, however, failure to characterise soft tissue contrast and the lack of molecular information is its limitation. Whole-body MRI is promising due to the lack of ionising radiation and the detailed imaging obtained of the soft tissues. It is also very good in determining metastasis to bone marrow, nodes, viscera and local soft tissues. However, it needs appropriate protocols and sequencing and expertise in analysis. Although recently, reporting standards were published, more work is needed to assess its role, as current evidence suggests that MRI is inferior to PET modalities.^7^ Of the PET modalities, ^68^Ga-PSMA and ^18^F-PSMA are increasingly employed. In a recent systematic review and meta-analysis, Perera et al^5^ reviewed the role of ^68^Ga-PSMA in prostate cancer. The authors reviewed 37 articles comprising 4790 patients and found positive percentage detection rates of 33%, 45%, 59%, 75%, and 95%, respectively with corresponding PSA categories of 0–0.19, 0.2–0.49, 0.5–0.99, 1–1.99, and ≥2 ng ml^−1^. Whilst this is the case, another recent systematic review and meta-analysis, showed that detection rate for ^18^F-PSMA was 86% with PSA of more than or equal to 0.5 ng ml^−1^ and 49% when PSA was less than 0.5 ng ml^−1^ for BRC.^8^ This shows the diagnostic utility of ^18^F-PSMA. The review also showed that ^18^F-PSMA can detect distant metastasis to lymph node and bone.^8^ This highlights that both PSMA PET modalities have high detection rates at lower PSA levels, and are comparable.

Despite excellent sensitivities of PSMA PET modalities, it is noteworthy that PSMA expression is not specific to the prostate. A variety of normal tissues express PSMA with intense uptake noted in salivary glands, lacrimal glands, the liver, spleen, pancreas, small intestine, bladder and renal cortex. As well as this physiological uptake, a number of other benign pathologies have been shown to express PSMA. Galiza Barbosa et al,^4^ in their recent review have summarised all the benign, malignant and inflammatory conditions showing increased uptake by ^68^Ga PSMA. Until recently, only a handful of case reports highlighted non-prostatic uptake of ^18^F-PSMA (Table 1). More recently, Rauschner et al ^14^ published a study assessing non-tumour uptake of PSMA ligands and compared ^68^Ga PSMA with ^18^F-PSMA. The authors found that ^18^F-PSMA has a fivefold higher incidence of benign non-tumour uptake compared to ^68^Ga PSMA. Predominant sites of non-tumour uptake with ^18^F-PSMA were ganglia (43%), lymph nodes (31%), bone (24%) and soft tissues (2%). Of the bone lesions, predominant site was ribs. The median SUV_max_ with ^18^F-PSMA for benign lesions was 5.3 (range 3.0–42.7) and for suspected tumour was 9.4 (range 2.7–234.4). Afaq et al,^15^ at the Society of Nuclear Medicine Annual meeting, presented results of their study that reviewed benign bone marrow uptake in ^18^F-PSMA using PET/MRI. In their study, lesions of marrow uptake with suspicious features on MRI were considered metastasis whereas lesions without corresponding MRI features were considered benign. They found that 7 of 34 (20%) patients had benign focal uptake.

**Table 1.** Non-prostatic uptake of ^18^F-18 PSMA

Authors (year)	Disease	Pattern of uptake
Pianou^9^	Paget’s disease	Diffuse uptake with SUVmax 9.
Marafi^10^	Glioma recurrence	
Marafi^11^	Brain metastasis from breast cancer	Intense uptake correlating well with MR.
Tang^12^	Hurthle cell thyroid adenoma	SUVmax 7.7
Haemels M^13^	Meningioma	Moderately PSMA-avid nodule

PSMA, prostate-specific membrane antige; SUVmax, maximum standardised uptake value.

Myeloma was detected on ^68^Ga PSMA,^16,17^ however, to our knowledge this is the first report of uptake of ^18^F PSMA-1007 in myeloma. Our case report illustrates a potential pitfall when imaging patients with ^18^F PSMA-1007 and adds to the growing body of literature of non-prostatic uptake of PSMA and highlights the need for reporters to be aware of this uptake. It is suggested that low positron energy of ^18^F, along with a higher signal of ^18^F PSMA-1007 (due to higher affinity and internalisation rate) and longer half-life may be attributable for having more benign uptake.^14^ The study from Afaq et al showed that MRI may be used to differentiate between benign and malignant marrow uptake.^15^

Although, prostate metastasis was considered as a possibility in our patient, it would be unusual as prostate metastases are usually sclerotic. So, emphasis was placed on alternate pathology such as myeloma which led the clinicians to perform appropriate tests. This enabled the clinicians to establish the diagnosis of myeloma based on serum electrophoresis and biopsy. Another feature in our patient was the diffuse uptake in the bone marrow contrary to the focal lesions noted by Afaq et al. In a recent report, myeloma was detected with ^68^Ga PSMA but they were discrete lesions.^17^ Therefore, more studies/reports are needed to evaluate whether this diffuse uptake is physiological or could be considered a suggestive feature of myeloma on ^18^F-18 PSMA.

## Conclusion

^18^F PSMA PET/CT is a rapidly expanding area of prostate imaging. Our case, of myeloma demonstrated on ^18^F-PSMA PET/CT is the first, to our knowledge, and illustrates that PSMA is not specific to the prostate and a range of benign and malignant pathologies may demonstrate PSMA uptake. It is important that reporters are aware of the biological distribution of these new tracers, which can differ between different ^18^F PSMA tracers and also beware of the potential pitfalls and uptake by other tumours.

## Learning points

1. PSMA uptake is not specific to prostate.
2. Non-prostatic pathological uptake is a potential pitfall of PSMA PET/CT imaging, and therefore reporting radiologists should be cautious about atypical uptake.
3. Non-sclerotic bone lesions should raise the suspicion of alternate pathologies.

## Consent

The authors confirm that written informed consent was obtained from the patient for publication of this case report, including accompanying images.
